# ELDER ABUSE SCREENING PRACTICES IN THE VETERANS HEALTH ADMINISTRATION: RESULTS FROM A NATIONWIDE EVALUATION

**DOI:** 10.1093/geroni/igac059.2755

**Published:** 2022-12-20

**Authors:** Lena Makaroun, Jaime Halaszynski, Kathrine Smith, Melissa Dichter, Ann-Marie Rosland, Carolyn Thorpe, Tony Rosen

**Affiliations:** VA Pittsburgh Healthcare System/University of Pittsburgh, Pittsburgh, Pennsylvania, United States; VA Butler Health Care System, Butler, Pennsylvania, United States; VA Fargo Health Care System, Fargo, North Dakota, United States; Temple University College of Public Health, Philadelphia, Pennsylvania, United States; University of Pittsburgh/VA Pittsburgh Healthcare System, Pittsburgh, Pennsylvania, United States; UNC Eshelman School of Pharmacy, Chapel Hill, North Carolina, United States; Weill Cornell Medical College / NewYork-Presbyterian Hospital, Pelham, New York, United States

## Abstract

Elder abuse (EA) is common and has devastating health consequences yet is rarely detected by healthcare professionals. Veterans are at high risk for EA, and the Veterans Health Administration (VHA) has experience screening for complex psychosocial phenomena including intimate partner violence. While the VHA has national policy regarding mandatory reporting of EA cases, little is known about the extent to which VHA sites currently screen for EA in a standardized fashion and what approaches are used. To address this knowledge gap, we conducted a national survey of all 170 parent station VHA medical centers from January to August of 2021. Surveys were distributed electronically to the Social Work Chief at each site, as social work is responsible for interpersonal violence response in VHA. The survey assessed the presence and characteristics of EA-specific screening practices as well as general abuse/neglect screening conducted with patients of all ages, including older adults. Follow up emails were sent to sites who reported conducting screening requesting additional details not included in the initial survey. Overall, 138 sites (81%) responded to the survey. Among respondents, 3% reported screening older adults for EA using a previously published tool, while 2% reported screening for EA with an unstudied or locally-developed tool. Forty-three percent reported doing general abuse/neglect screening using unstudied questions/tools for patients of all ages, and 41% reported no EA screening at their site. The wide variability in current EA screening practices in VHA presents an important opportunity to standardize and improve EA detection practices.

